# The impact of daily temperature on renal disease incidence: an ecological study

**DOI:** 10.1186/s12940-017-0331-4

**Published:** 2017-10-27

**Authors:** Matthew Borg, Peng Bi, Monika Nitschke, Susan Williams, Stephen McDonald

**Affiliations:** 1 School of Public Health, University of Adelaide, Adelaide, South Australia 5005 Australia; 2 SA Health, Government of South Australia, Adelaide, South Australia Australia; 3 The Central Northern Renal and Transplantation Service, Royal Adelaide Hospital, Adelaide, South Australia Australia

**Keywords:** Temperature, Heat, Renal disease, Urolithiasis, Acute kidney injury, Renal failure, Chronic kidney disease, Urinary tract infections, Lower urinary tract infections, Pyelonephritis

## Abstract

**Background:**

Extremely high temperatures over many consecutive days have been linked to an increase in renal disease in several cities. This is becoming increasingly relevant with heatwaves becoming longer, more intense, and more frequent with climate change. This study aimed to extend the known relationship between daily temperature and kidney disease to include the incidence of eight temperature-prone specific renal disease categories – total renal disease, urolithiasis, renal failure, acute kidney injury (AKI), chronic kidney disease (CKD), urinary tract infections (UTIs), lower urinary tract infections (LUTIs) and pyelonephritis.

**Methods:**

Daily data was acquired for maximum, minimum and average temperature over the period of 1 July 2003 to 31 March 2014 during the warm season (October to March) in Adelaide, South Australia. Data for daily admissions to all metropolitan hospitals for renal disease, including 83,519 emergency department admissions and 42,957 inpatient admissions, was also obtained. Renal outcomes were analyzed using time-stratified negative binomial regression models, with the results aggregated by day. Incidence rate ratios (IRR) and 95% confidence intervals (CI) were estimated for associations between the number of admissions and daily temperature.

**Results:**

Increases in daily temperature per 1 °C were associated with an increased incidence for all renal disease categories except for pyelonephritis. Minimum temperature was associated with the greatest increase in renal disease followed by average temperature and then maximum temperature. A 1°C increase in daily minimum temperature was associated with an increase in daily emergency department admissions for AKI (IRR 1.037, 95% CI: 1.026–1.048), renal failure (IRR 1.030, 95% CI: 1.022–1.039), CKD (IRR 1.017, 95% CI: 1.001–1.033) urolithiasis (IRR 1.015, 95% CI: 1.010–1.020), total renal disease (IRR 1.009, 95% CI: 1.006–1.011), UTIs (IRR 1.004, 95% CI: 1.000–1.007) and LUTIs (IRR 1.003, 95% CI: 1.000–1.006).

**Conclusions:**

An increased frequency of renal disease, including urolithiasis, acute kidney injury and urinary tract infections, is predicted with increasing temperatures from climate change. These results have clinical and public health implications for the management of renal diseases and demand tailored health services. Future research is warranted to analyze individual renal diseases with more comprehensive information regarding renal risk factors, and studies examining mortality for specific renal diseases.

**Electronic supplementary material:**

The online version of this article (10.1186/s12940-017-0331-4) contains supplementary material, which is available to authorized users.

## Background

Excessive seasonal temperatures predispose to adverse heat-related human morbidity in multiple organ systems [1, 2]. These health effects have mostly been investigated with regards to cardiovascular, respiratory and well-known heat-related diseases [3], but have also been observed for renal disease [1, 3–6]. This is becoming increasingly more relevant due to the impact of climate change [7–9]. Two studies in Adelaide, Australia, showed increased admissions for renal disease during heatwaves through all ages, by 10% and 13%, respectively [10, 11], and another study in Adelaide also identified an association between temperature and renal disease [12]. International studies have shown similar results [3, 13–15]. Studies in the United States of America (USA) linked incremental rises in same-day apparent temperatures to an increased occurrence of renal disease and acute kidney injury (AKI, also known as acute renal failure) [1, 13].

In many cities, high temperatures have been linked to an increased occurrence of AKI [3, 11, 13, 15–17] and chronic kidney disease (CKD, also known as chronic renal failure) [18, 19]. A study in Sydney, Australia, reported that the incidence of AKI and renal failure (AKI and CKD combined) increased if the daily temperature was above the 95th percentile range for one day, and this further increased if the high temperature persisted for three days [4]. AKI and urolithiasis also predispose to chronic kidney disease, though paradoxically, CKD can help prevent urolithiasis due to a disease-associated decrease in urinary calcium secretion [20, 21].

The risk of renal colic (pain due to urolithiasis) increases with higher daily temperature [22–26]. Studies in Australia and New Zealand have demonstrated seasonal variation in the incidence of renal colic, highest in summer and autumn [8, 27, 28]. A USA study also reported that the incidence of urolithiasis is greatest in warmer areas, specifically uric acid and calcium stones [14]. Even though urinary stones usually form over months [25, 29], investigations in Sydney and the USA showed the risk for urolithiasis was highest ≤3 days after exposure to high ambient temperatures, with a peak at day one [4, 13, 30]. The risk can be increased for the next 20 days as well [25]. It has been suggested that the incidence and prevalence of urolithiasis are increasing due to climate change [14, 31].

Research on the relationship between ambient air temperature and urinary tract infections (UTIs) is inconclusive. UTIs include pyelonephritis (infection of renal pelvis, calyces and/or tissue) and lower urinary tract infections (LUTIs) which are UTIs occurring below the renal pelvis. One trial in the USA found a positive association between hospitalizations during heatwaves and primary diagnoses for UTIs and septicemia [3]. Other studies have not found such association [4]. Hospitalizations and ED admissions for nephritis increased significantly during the 2006 California heatwave [17].

Whilst studies in different countries have examined these various renal outcomes, their results might not be generalizable to countries with different climate and population characteristics [32, 33]. A previous study in Adelaide, Australia, found increased incidence of AKI and renal disease during heatwaves [11]. The present study extends those findings by considering a wider range of specific renal diseases and their associations with temperature. It is the first study to investigate the link between heat exposure and urolithiasis, UTIs and CKD in an Australian population. The results will have clinical and public health implications for the management of renal diseases in a temperate climate region.

## Methods

### Aim and design

To examine if the incidence of eight temperature-prone specific renal disease categories in metropolitan Adelaide would increase in relation to a 1°C increase in daily dry bulb temperature. This study is an ecological study with a time-series approach.

### Setting

Adelaide’s predicted population in June 2016 was 1.32 million [34]. With a total land area of 983,482 km^2^ [35], the state has a semi-arid climate with mild winters and dry, hot summers with cool nights [36]. Adelaide recently experienced multiple heatwaves including during March 2008, January to February 2009, November 2009 and during the summer of 2013–2014 [37]. 2013 was the warmest year on record for Adelaide, and 2015 was the second warmest [38]. Increased frequency, intensity and duration of hot days are predicted in Australia with climate change [39].

### Data collection

Hospital inpatient and ED admissions data for a range of International Classification of Diseases, 10th revision (ICD-10) classification codes were acquired from the South Australian Department of Health for the period spanning 1 July 2003 to 31 March 2014. Data represented admissions to hospitals within the Adelaide metropolitan region. Elective admissions (patient did not require admission within 24 h) were excluded from the data because emergency admissions (patient required admission within 24 h) are more likely to reflect the acute effects of heat exposure.

Daily meteorological data for maximum and minimum dry bulb temperature were obtained from the Bureau of Meteorology from a monitoring station in Kent Town (station number 023090) [37], Adelaide. Data from this station is representative of climate across the Adelaide metropolitan area. Because this data only represented the metropolitan area, admission of patients living in rural areas were excluded from this study. Daily maximum and minimum temperatures were recorded at 9 am; the data is included in Additional file 1. The highest temperature recorded in the 24 h prior to 9 am is recorded as the maximum daily temperature for the previous day which usually occurs in the afternoon, while the lowest temperature occurring in the same 24 h is recorded as the daily minimum temperature for the current day which usually occurs overnight or near dawn [40]. The average daily temperature was calculated from these daily maximum and minimum temperatures. Descriptive statistics of the temperature data is included in Table 1.

**Table 1:** Descriptive statistics for temperature

Temperature	Mean	SD	5th percentile	95th percentile	Median
Maximum	27.4	6.26	18.2	38.7	26.7
Minimum	15.54	4.48	8.8	23.7	15
Average	21.47	4.94	14.1	30.85	20.85

Descriptive statistics for daily dry bulb maximum, minimum and average temperature in Adelaide from 1 July 2003 to 31 March 2014 excluding the warm season (October – March)

Diseases were classified according to the ICD-10 codes. Admissions data were accessed for records with a primary diagnosis of renal disease (N00 – N39). Primary diagnoses were obtained for both inpatient and ED admissions, and also up to two secondary diagnoses were obtained for inpatient admissions. Secondary diagnoses could not be obtained for ED admissions due to South Australian (SA) Department of Health protocol. Admissions were then further classified on specific primary and/or secondary diagnoses for eight renal diseases. These diseases were urolithiasis (N20 – N23), renal failure (N17 – N19.9), AKI (N17.0–17.9), CKD (N18.1 – N18.9), UTIs (N10 – N12, N30, and N39.0–39.2), LUTIs (N30; N39.0 – N39.2), and pyelonephritis (N10 – N12). UTIs were investigated (i) collectively (pyelonephritis and LUTIs) to enable comparison with previous studies that did not sub-classify UTI based on the site of infection [3, 41] and (ii) separately to provide more detailed information. Similarly, both AKI and CKD were investigated both separately and collectively, as renal failure, for comparison with previous studies that grouped AKI and CKD together [30].

### Data analyses

Negative binomial (NB) regression models were used to estimate incidence rate ratios (IRRs) for daily admissions for various renal diseases (outcome variables) per 1°C increase in maximum, minimum and average temperature. Negative binomial models were deemed appropriate because of the overdispersion in the data. The models were adjusted for the effects of year, month, day of the week and holidays by including categorical variables for each covariate, and the outcomes were aggregated by day. Separate regression models were generated for inpatient and ED admissions. Models were time-stratified including only admissions occurring within the warm season (October to March), because the focus of the study was heat-related renal morbidity; including the cold season could introduce the confounding effect of cold on renal disease incidence [11, 12, 42, 43]. Years were assigned from 1 July to 30 June (financial years) to ensure continuity across successive warm seasons. Secondary analyses were performed to (i) examine renal admissions following a lag period of 1–5 days after daily temperatures, (i) repeat the primary analysis with further stratification by gender, and (iii) repeated the primary analysis with stratification by age instead of gender (<65 years and ≥65 years old). All statistical analyses were conducted using Stata v13.0 (Statacorp, Texas, USA) with a significance level of *P* < 0.05.

NB models were used because the outcome variables were counts, and these models are appropriate for overdispersed data. Goodness-of-fit tests were undertaken to indicate if Poisson models, the standard model of choice for count variables, fit the data [44]. These tests indicated a poor fit, as shown in Appendix 1. As the data contains very few zero values, the poor fit was likely secondary to overdispersion, justifying the use of NB models.

The primary statistical models can be represented by the equation:

$$ {\log}_{\mathrm{e}}\ \left({\mathrm{N}}_{\mathrm{renal}}\right)={\upbeta}_0+{\upbeta}_1\mathrm{temp}+{\upbeta}_2\mathrm{holiday}+{\sum}_{0\le \mathrm{m}\le 5}\left({\upbeta}_{3+\mathrm{m}}{\mathrm{m}\mathrm{onth}}_{\mathrm{m}}\right)+{\sum}_{0\le \mathrm{n}\le 6}\left({\upbeta}_{9+\mathrm{n}}{\mathrm{day}}_{\mathrm{n}}\right)+{\sum}_{0\le \mathrm{o}\le 10}\left({\upbeta}_{16+\mathrm{o}}{\mathrm{year}}_{\mathrm{o}}\right)+\mathrm{u} $$

*N* designates the expected number of admissions for the specified renal disease *renal*. *Temp* is a variable representing daily temperature across all the days included in the study, specified as either maximum, minimum or average temperature. *β*_*1*_ is the regression coefficient representing the effect of a 1°C increase in *temp*. Exp(*β*_*1*_) is the calculated IRR for an increase in admissions per 1°C increase in temperature. In short, *β*_*1*_ *temp* represents the effect of temperature. Similarly *β*_*2*_ *holiday* represents the effect of whether the day was a public holiday. *β*_*3 + m*_ *month*_*m*_ represents the effect of the month; using a categorical variable for each month (*m*) from October to March. Similarly, *β*_*9 + n*_ *day*_*n*_ represents the effect of the day of the week, using a categorical variable for each day of the week (*n*). *β*_*16 + o*_ *year*_*o*_ represents the effect of the year, using a categorical variable for each financial year (o) within this study. *β*_*0*_ and *u*_*t*_ represent estimated constants in the model; u_t_ is specifically included in NB models to account for extra variance.

## Results

A total of 3927 days were included in the study period, with a total of 83,519 ED and 42,957 inpatient admissions for renal disease. Table 2 provides summary statistics for daily incidence of inpatient and ED admissions for specific renal diseases, showing positively skewed distributions for renal admissions. Mean daily admissions for renal diseases during the warm season were 11.5 for inpatient and 22.4 for ED admissions.

**Table 2:** Summary statistics for admissions for renal disease

Description	Observational time period (days)	Daily incidence of admissions								Total admissions	
		ED				Inpatient				ED	Inpatient
		Mean	SD	Min Max		Mean	SD	Min Max			
Total renal	Total: 3927	21.27	6.04	5	46	10.94	4.25	1	32	83,519	42,957
disease	Cold season: 1922	20.26	5.53	5	42	10.32	3.90	1	28	38,931	19,834
	Warm season: 2005	22.24	6.35	8	46	11.53	4.48	1	32	44,588	23,123
Urolithiasis	Total: 3927	4.88	2.52	0	18	2.09	1.54	0	10	19,171	8206
	Cold season: 1922	4.38	2.23	0	15	1.86	1.44	0	9	8409	3578
	Warm season: 2005	5.37	2.69	0	18	2.31	1.60	0	10	10,762	4628
Renal failure	Total: 3927	1.79	1.53	0	13	1.84	1.53	0	13	7024	7213
	Cold season: 1922	1.70	1.46	0	7	1.75	1.47	0	8	3263	3359
	Warm season: 2005	1.88	1.58	0	13	1.92	1.58	0	13	3761	3854
AKI	Total: 3927	1.08	1.19	0	10	1.40	1.34	0	12	4253	5483
	Cold season: 1922	0.99	1.08	0	6	1.29	1.24	0	7	1904	2481
	Warm season: 2005	1.17	1.27	0	10	1.50	1.42	0	12	2349	3002
CKD	Total: 3927	0.50	0.73	0	5	0.86	0.99	0	6	1959	3390
	Cold season: 1922	0.51	0.75	0	5	0.85	0.99	0	5	976	1634
	Warm season: 2005	0.49	0.70	0	4	0.88	0.98	0	6	983	1756
UTI	Total: 3927	13.70	4.39	2	33	6.36	2.92	0	19	53,789	24,985
	Cold season: 1922	13.30	4.19	2	31	6.12	2.75	0	19	25,559	11,754
	Warm season: 2005	14.08	4.55	2	33	6.60	3.05	0	19	28,230	13,231
Lower UTI	Total: 3927	12.27	3.97	1	31	5.35	2.64	0	15	48,203	20,997
	Cold season: 1922	11.97	3.80	1	26	5.19	2.49	0	14	23,004	9978
	Warm season: 2005	12.57	4.11	2	31	5.50	2.76	0	15	25,199	11,019
Pyelonephritis	Total: 3927	1.42	1.34	0	9	1.21	1.13	0	7	5586	4755
	Cold season: 1922	1.33	1.29	0	9	1.10	1.07	0	6	2555	2123
	Warm season: 2005	1.51	1.38	0	9	1.31	1.17	0	7	3031	2632

Summary statistics for daily admission rates and total admissions for renal diseases in Adelaide from 1 July 2003 to 31 March 2014. Admissions are divided into cold season (April – September) and warm season (October – March). Both emergency department (ED) and inpatient admissions are included. Accompanying the mean daily admission rate (mean) are the standard deviation (SD) and the minimum (min) and maximum (max) number of daily admissions

**Fig. 1:**
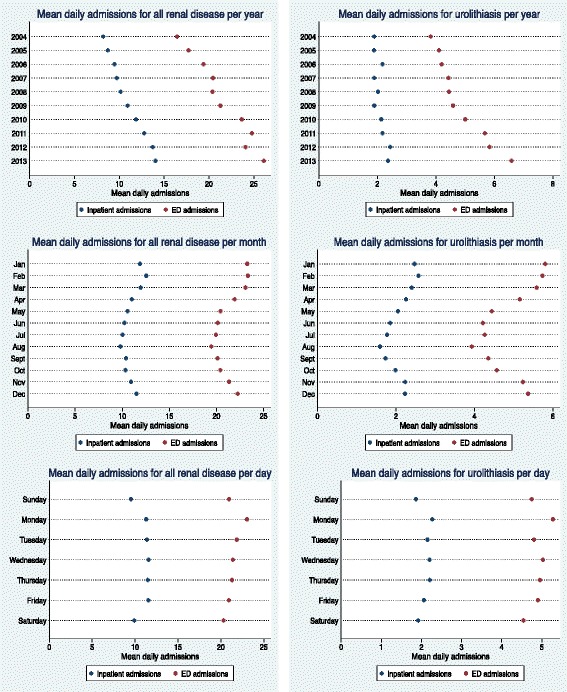
Mean daily renal admissions by year, month and day. Mean daily admissions for total renal disease and urolithiasis plotted against year, month and day of the week in Adelaide. Both inpatient and emergency department (ED) admissions were included. Holidays were excluded from the data. The time period illustrated in the dot graphs examining month and day of the week was 1 July 2003–31 March 2014

**Fig. 2:**
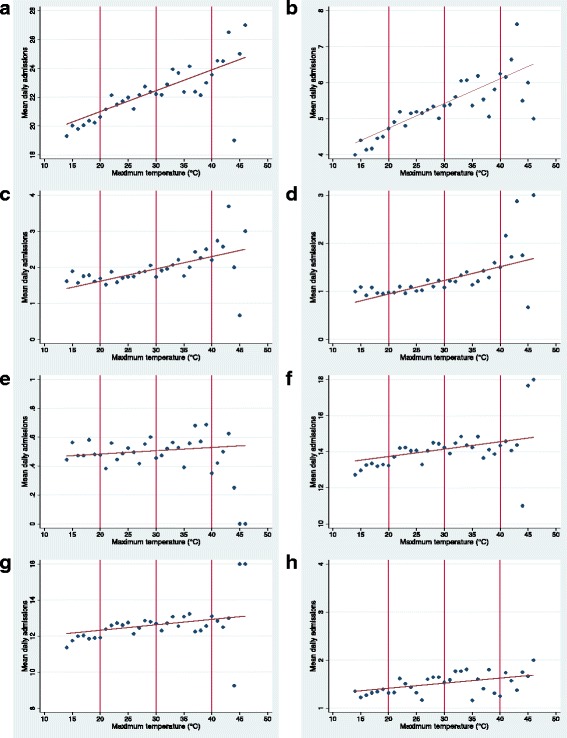
Daily maximum temperature and mean hospital emergency department renal admissions. Descriptive graphs for mean number of daily hospital emergency department admissions for renal disease in relation to daily maximum temperature, during the warm season (October – March) in Adelaide from 1 July 2003 to 31 March 2014. Data points are separated by 1°. Graphs are in relation to (**a**) total renal disease; (**b**) urolithiasis; (**c**) renal failure; (**d**) AKI; (**e**) CKD; (**f**) UTI; (**g**); lower UTI; (**h**) pyelonephritis

**Fig. 3:**
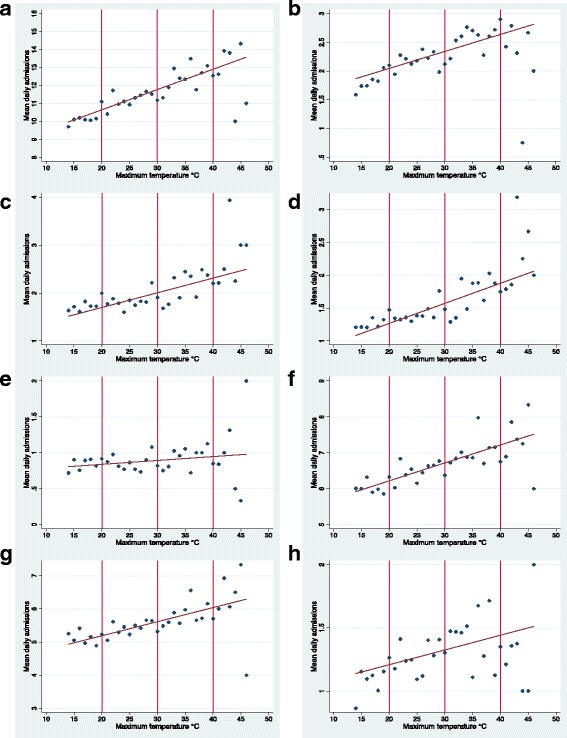
Daily maximum temperature and mean hospital inpatient renal admissions. Descriptive graphs for mean number of daily hospital inpatient admissions for renal disease in relation to daily maximum temperature rounded to the nearest 1°C during the warm season (October – March) in Adelaide from 1 July 2003 to 31 March 2014. Data points are separated by 1°. Graphs are in relation to (**a**); total renal disease (**b**); urolithiasis (**c**); renal failure (**d**); AKI (**e**); CKD (**f**); UTI (**g**); lower UTI (**h**); pyelonephritis

Figure 1 shows the incidence of mean daily admissions for all renal disease and urolithiasis plotted against year, month and day of the week. Admissions were highest during the warmest months (December – March) and from Monday to Friday. Mean daily admissions for renal failure, AKI, CKD, UTIs, LUTIs and pyelonephritis showed similar trends in relation to time (data not shown). Mean daily admissions for renal diseases were plotted against daily maximum temperature rounded to the nearest degree for ED and inpatient admissions (Fig. 2 and Fig. 3 respectively). The relationships between admissions and temperature appeared linear and positive as evidenced by the line of best fit. Stronger relationships with temperature were apparent for total renal disease (a), urolithiasis (b), renal failure (c) and AKI (d), with weaker relationships apparent for the other renal categories. The results between ED and inpatient admissions were similar apart from a greater percentage of increase in UTI and LUTI inpatient admissions compared to ED admissions. At very high temperatures (starting at approximately 40°C), there were fewer observations for daily admission counts; this showed as greater scattering and the presence of outliers. These outliers are likely to have had minimal impact on estimating the effect of temperature because they only represented a very small number of days.

**Fig. 4:**
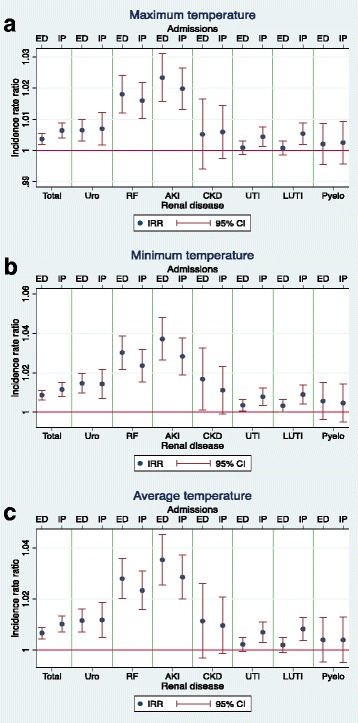
Estimated incidence rate ratios (IRRs) for renal outcomes and daily temperature. Graphs for incidence rate ratios (IRRs) for daily admissions for renal diseases and associated 95% confidence intervals (95% CI) in relation to daily temperature per 1°C, during the warm season (October – March) in Adelaide from 1 July 2003 to 31 March 2014. Both emergency department (ED) and inpatient (IP) admissions are included. Graphs are in relation to (**a**); maximum temperature (**b**); minimum temperature (**c**); average temperature. The renal categories represented are total renal disease (Total), urolithiasis (Uro), renal failure (RF), acute kidney injury (AKI), chronic kidney disease (CKD), urinary tract infections (UTI), lower urinary tract infections (LUTI) and pyelonephritis (Pyelo)

Table 3 shows estimated IRRs for each renal disease outcome in relation to a 1°C increase in daily maximum, minimum and average temperature for inpatient and ED admissions. A positive relationship was identified for total renal disease, urolithiasis, renal failure and AKI for both inpatient and ED admissions, as well as for UTI and LUTI for inpatient admissions. Minimum temperature (but not maximum and average temperature) was also associated with statistically significant increase for ED admissions for CKD, UTI and LUTI. Minimum temperature appeared to be associated with the greatest increase followed by average temperature. The highest effect was apparent for admissions for AKI. There was a 3.7% increase in ED admissions for AKI for each 1°C increase in minimum temperature (IRR = 1.037, 95% CI 1.026–1.048 *P* < 0.001). A comparable effect was also observed for renal failure for both ED and inpatient admissions in association with 1°C increases in average and minimum temperature, with the other outcomes either showing a smaller increase or no significant effect. Pyelonephritis admissions showed no association with any of the temperature metrics. These results are also shown graphically in Fig. 4.

**Table 3:** Regression analysis for temperature

Temperature data	Admission data	Disease	IRR	95% CI			*P*-value
Maximum	ED	All renal disease	1.004	1.002	–	1.005	<0.001
		Urolithiasis	1.006	1.003	–	1.010	<0.001
		Renal failure	1.018	1.012	–	1.024	<0.001
		AKI	1.023	1.016	–	1.031	<0.001
		CKD	1.005	0.994	–	1.016	0.365
		UTI	1.001	0.999	–	1.003	0.411
		Lower UTI	1.001	0.999	–	1.003	0.507
		Pyelonephritis	1.002	0.995	–	1.009	0.547
	Inpatient	All renal disease	1.006	1.004	–	1.009	<0.001
		Urolithiasis	1.007	1.002	–	1.012	0.009
		Renal failure	1.016	1.010	–	1.022	<0.001
		AKI	1.020	1.013	–	1.026	<0.001
		CKD	1.006	0.997	–	1.014	0.171
		UTI	1.004	1.001	–	1.008	0.005
		Lower UTI	1.005	1.002	–	1.009	0.002
		Pyelonephritis	1.002	0.996	–	1.009	0.472
Minimum	ED	All renal disease	1.009	1.006	–	1.011	<0.001
		Urolithiasis	1.015	1.010	–	1.020	<0.001
		Renal failure	1.030	1.022	–	1.039	<0.001
		AKI	1.037	1.026	–	1.048	<0.001
		CKD	1.017	1.001	–	1.033	0.036
		UTI	1.004	1.000	–	1.007	0.023
		Lower UTI	1.003	1.000	–	1.006	0.045
		Pyelonephritis	1.006	0.996	–	1.015	0.245
	Inpatient	All renal disease	1.012	1.008	–	1.015	<0.001
		Urolithiasis	1.014	1.007	–	1.022	<0.001
		Renal failure	1.024	1.015	–	1.032	<0.001
		AKI	1.028	1.019	–	1.038	<0.001
		CKD	1.011	0.999	–	1.023	0.068
		UTI	1.008	1.003	–	1.012	<0.001
		Lower UTI	1.009	1.004	–	1.014	<0.001
		Pyelonephritis	1.005	0.995	–	1.014	0.346
Average	ED	All renal disease	1.007	1.004	–	1.009	<0.001
		Urolithiasis	1.012	1.007	–	1.016	<0.001
		Renal failure	1.028	1.020	–	1.036	<0.001
		AKI	1.035	1.025	–	1.045	<0.001
		CKD	1.011	0.997	–	1.026	0.125
		UTI	1.002	0.999	–	1.005	0.121
		Lower UTI	1.002	0.999	–	1.005	0.184
		Pyelonephritis	1.004	0.995	–	1.013	0.364
	Inpatient	All renal disease	1.010	1.007	–	1.013	<0.001
		Urolithiasis	1.012	1.005	–	1.019	0.001
		Renal failure	1.023	1.016	–	1.031	<0.001
		AKI	1.029	1.020	–	1.037	<0.001
		CKD	1.010	0.999	–	1.021	0.087
		UTI	1.007	1.003	–	1.011	0.001
		Lower UTI	1.008	1.004	–	1.013	<0.001
		Pyelonephritis	1.004	0.995	–	1.013	0.378

Estimated incidence rate ratios (IRRs) and associated 95% confidence intervals and *P*-values for daily admissions for renal diseases in relation to an increase in daily temperature (maximum, minimum and average) per 1°C during the warm seasons (October – March) in Adelaide from 1 July 2003 to 31 March 2014. Both emergency department (ED) and inpatient admissions were included

Incidence for daily admissions for renal disease in relation to a 1°C increase in daily temperature (maximum, minimum and average) for admissions for all the renal categories following a lag period of 1–5 days were examined. The results show a lag effect of temperature was associated with all the renal categories and daily temperature (maximum, minimum and average) with the exception of pyelonephritis. This lag effect appeared to last the longest for total renal disease, urolithiasis, renal failure and AKI. Admissions for CKD were associated with a lag effect even if admission were not increased on the day when the daily temperatures were recorded. Similar to the admissions rates without a lag period, IRRs were generally highest when associated with minimum daily temperature followed by average temperature and then maximum temperature. The effect of minimum temperature appeared to be strongest on the day as opposed to following a lag period, with the exception of a very small increase in IRR for CKD for both IP and ED admissions (0.1%). When examining maximum temperature, however, the effect appeared to be strongest 1–3 days after the temperature exposure. The lag period analyses, and accompanying graphs for maximum and minimum temperature, are shown in Appendix 2.

The effects of gender on the associations between renal admissions and temperature were examined; however, there were less statistically relevant results when looking at genders separately, likely due to reduced power. For male ED presentations, results were statistically significant for total renal disease and urolithiasis. For male inpatient presentations, results were statistically significant for AKI and CKD instead. For ED presentations among females, results were statistically significant for renal failure, AKI, UTIs and LUTIs. The only statistically significant changes in female inpatient presentations were with admissions for urolithiasis. Both genders had increased ED admissions for total renal disease. Males also showed increased ED admissions for urolithiasis and renal failure, whilst females demonstrated increased ED admissions for renal failure, AKI, UTIs and LUTIs. The gender-stratified analyses, including graphs, are available in Appendix 3.

The effects of age group (<65 years and ≥65 years) on the associations between renal admissions and temperature were examined. In those aged <65 years old, there were statistically significant increases in ED admissions for total renal disease, urolithiasis, renal failure, and AKI associated with daily temperature (maximum, minimum and average). For elderly-related admissions, the only statistically significant increase in inpatient admissions was for pyelonephritis. The age-stratified analyses, including graphs, are available in Appendix 4.

## Discussion

This study investigated the associations between temperature and admissions for a range of specific renal diseases in Adelaide, a city with a temperate climate. No previous Australian study has investigated the link between heat exposure and urolithiasis, UTIs and CKD at a population level. Furthermore it is the only SA study that examines the effect of lag periods and gender on heat exposure with regards to renal disease. The only previous SA study in Adelaide that investigated a direct link between temperature and renal disease, only assessed the relationship above predefined temperature thresholds, and it did not investigate specific renal categories [12].

This study showed an increased incidence of renal disease, urolithiasis, renal failure (both acute and chronic) and UTIs, specifically lower UTIs, in relation to daily temperature (maximum, minimum and average). Inpatient and ED admissions for total renal disease, renal failure and AKI increased with daily maximum, minimum and average temperatures, and ED admissions for CKD increased with minimum temperature. The results for total renal disease and AKI are consistent with previous studies in Adelaide and Sydney [4, 10, 12, 43]. An increase was also found in inpatient admissions for UTIs and LUTIs in association with increasing temperature. Older patients are normally more prone to UTIs, and concurrent dehydration stress could predispose them further [41]. We did not find a significant increase in UTIs in the population aged over 65 years, however this may have been due to a decrease in statistical power because of sample size. ED and inpatient admission rates for urolithiasis increased with higher temperatures. Australian studies have demonstrated seasonal variation in the incidence of renal colic – highest in summer and autumn [27, 28, 45], which is consistent with our findings. Admissions for AKI showed the greatest increase in relation to heat exposure with maximum, minimum and average daily temperature. Dehydration has a stronger established link with AKI [46]. Comparable effects of temperature on renal failure (AKI and CKD collectively) were observed, which were likely due to the increased AKI admissions. On a similar note, as the large majority of total renal disease cases were AKI and urolithiasis, these admissions are likely to explain the increased admissions for total renal disease with heat exposure.

**Fig. 5:**
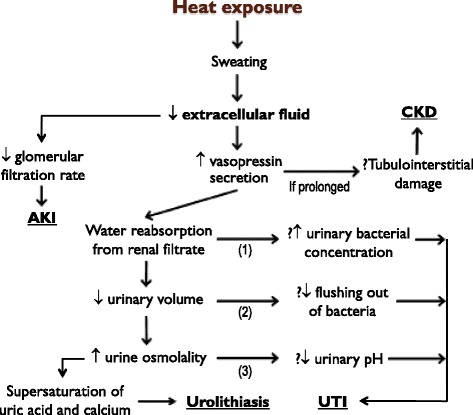
Proposed mechanisms for heat exposure leading to renal disease. Heat exposure can induce sweating, leading to decreased extracellular fluid (ECF) with subsequent dehydration. This can lead to renal disease by a variety of mechanisms [14, 41, 49]

High temperatures are believed to predispose to renal disease due to heat-induced sweating, leading to decreased extracellular fluid (ECF) and subsequent dehydration [14]. In the case of urolithiasis, low ECF leads to increased secretion of vasopressin. Vasopressin promotes reabsorption of water from renal filtrate causing increased urinary concentration. This increases the concentration of calcium and uric acid, predisposing to supersaturation of these solutes and hence accelerating the rate of stone formation [14, 25, 41, 47]. Stones can grow over hours in an appropriate urinary environment [48]. Low ECF, with subsequent decreased blood volume, results in less blood being filtrated by the kidney and hence a decreased glomerular filtration rate. This can lead to AKI [46, 49]. Studies have shown dehydration is linked to a higher risk of both AKI and CKD [45, 50]. Whilst there is evidence to suggest a positive association exists between dehydration and CKD, including an Australian study, a precise mechanism has not been established [41, 45, 50, 51]. A proposed mechanism is that prolonged elevated vasopressin secretion, induced by chronic dehydration, contributes to progressive tubulointerstitial damage, predisposing to CKD [41, 52]. A USA study showed increased admissions for UTIs during heatwaves [3]. Chronic low urine output, which can result from decreased urine volume secondary to dehydration, has been linked to a higher risk of UTI [41]. Hypothesized, but unproven, mechanisms for this observation stem from decreased water within the urine. This leads to increased bacterial concentration (predisposing to infection) and increased urine osmolality with subsequent urine acidity that promotes bacterial adhesion to the urinary epithelium [41]. Decreased urinary volume can also result in less “flushing out” of bacteria [41]. These mechanisms for dehydration resulting in renal disease are outlined in Fig. 5.

CKD was the only renal category in this study that required a lag period in order to indicate a statistically significant increase in admissions. This may reflect the chronic nature of CKD in which the clinical effect of worsening renal function secondary to dehydration does not present immediately but rather predisposes to an admission approximately 1–3 days later.

The effect of increasing temperature on ED admissions for urolithiasis was higher in males than females. This is consistent with the observation that men are more likely to develop calcium and uric stones and that stones can develop quickly following acute heat exposure [14, 47, 53]. This increased risk is amplified with increasing temperature. [14]. In contrast, the effect of increasing temperature on ED admissions for UTIs and LUTIs was only apparent in females. This was probably because females are at greater risk of developing UTIs; one study showed they are 50 times more frequent in adult women without accounting for heat exposure [41].

It is known that infants, children, and particularly the elderly, are more susceptible to heat-related morbidity than other age groups [4, 11, 17, 42, 54–56]. When examining the effect of age in this study, those aged <65 had statistically significant results for ED admissions for urolithiasis, renal failure and AKI, but not the elderly. This could have reflected the acute nature of dehydration-predisposing activities that those aged <65 undertake, such as intense exercise or severe occupational heat exposure, whereas the onset of dehydration in the elderly may be more gradual. Alternatively the difference between the results in the two age groups could have resulted from a lack of statistical power. It should be noted that there were less statistically relevant results when stratifying by genders and age. For example, inpatient admissions for UTIs and LUTIs were statistically significant for maximum, minimum and average temperature for both genders combined, but no significance was found when examining the genders individually.

Adverse health effects of extreme heat exposure are largely preventable [57]. The SA Government is undertaking prevention strategies to reduce morbidity during heat events. These strategies include media announcements raising awareness of heat events and advice to minimize heat-related morbidity [58]. Such advice includes maintaining adequate hydration, use of air conditioning, keeping out of the sun and cool clothing [58]. The increased risk of renal disease should also be advertised to the general public as part of these strategies. Certain individuals deemed to be more vulnerable to heat-related morbidity, including the elderly and some mental health clients, are targeted [4, 11, 17, 42, 54–56, 58]. The assessment and monitoring of vulnerable individuals is carried out by community health managers [58]. These vulnerable individuals are further targeted by these strategies, such as phone calls to these individuals or their carers for additional heat event awareness and advice [58]. People with a significantly high risk of developing renal disease, as identified by clinicians, should also be included in this vulnerable group of individuals, because renal disease is more likely to occur during heatwaves. For example, in the case of AKI, fluid-restricted individuals or those taking nephrotoxic medications [46], and in the case of urolithiasis, patients with a past history of urolithiasis [28]. This is especially important for AKI, as it is associated with increased mortality [59]. Primary care and renal physicians should inform vulnerable patients of the risks and recommend preventative measures during their consultations, particularly shortly before and throughout the warm season. Furthermore health service providers could tailor these risk-reducing strategies to each individual patient, for example, by recommending a patient to rehydrate at set times during the day with a set volume at each time, and to have extra hydration following prolonged heat exposure such as during work. At a policy level, government and service providers should consider increasing the capacity for renal health care, particularly during the warm season, in preparation for the increase in renal disease frequency.

This study provides the most comprehensive examination of different renal disease categories in relation to heat exposure to date in Australia. Over 80,000 ED admissions and 40,000 inpatient admissions were tested in this study; the large sample size is necessary to provide adequate statistical power when examining subdivided renal categories. Multiple measures of temperature were tested to add reliability to the results and lag periods of up to five days following temperature exposure were assessed. This study also considered differences between genders and age groups.

This study has some limitations, including potential misclassification of heat exposure. Those without access to air conditioning have increased heat exposure, as air conditioners are protective for heat adverse outcomes [1, 60–63]. However, Adelaide has high air-conditioning coverage, therefore its impact should be minimum. This study did not account for humidity and air pollution, in particular ozone and particulate matter, which have been linked to greater heat-related morbidity; but this effect is minimal, and air pollution may not affect renal disease [12, 17, 18, 45, 64, 65]. All temperature data were acquired from a single meteorological station, and, while this is representative of metropolitan Adelaide, it would have been more accurate to utilize data from multiple climate stations matched to the patients’ home addresses. However, other stations reporting meteorological data from metropolitan Adelaide, including Adelaide and Parafield Airports, showed similar temperatures to those of Kent Town [37]. When examining age, it would have been preferred to assess a number of age groups, such as children. However, the main concerning age, 65 and above (the elderly), was examined [4, 11, 17, 42, 55, 56]. With ICD-codes, diagnoses can be under-reported or miscoded [3, 11, 26]. Only up to two secondary diagnoses for inpatient admissions, and none for ED admissions, were included, hence the number of admissions were likely under-reported. Finally, medical complications attributed to heat events may occur at some time even without heat exposure; that is, the exposure would lead to an earlier complication instead of an additional one [3, 66, 67].

Further research is warranted to analyze individual renal diseases in more detail. To complement population based studies, a clinical audit trial could enable data to be collected from hospital records for targeted renal outcomes during heat events, revealing more comprehensive information regarding clinical, social and behavioral risk factors. These factors could include co-morbidities such as diabetes and heart failure that have been associated with worse outcomes during severe heat exposure [54, 68, 69]. Certain occupations, such as mining and agriculture, are risk factors due to occupational heat exposure, which is additive to seasonal exposure [5, 70–73]. Psychiatric conditions, impaired mobility, a greater number of co-morbidities, social isolation and low socio-economic status have been linked to adverse heat outcomes [16, 18, 19, 30, 62, 73, 74]. Medications that can increase susceptibility to heat-related renal disease by impairing thermotolerance, in particular psychotropic drugs, diuretics and β-blockers, should also be evaluated [11, 75–77]. The SA Extreme Heat Arrangements Plan, a SA government initiative which implements heat warning and targeted interventions during periods of extreme heat, has been shown to lower the rates of morbidity outcomes associated of renal disease [78]. This plan could be further evaluated by investigating its effectiveness on specific renal diseases.

## Conclusions

An increased risk of specific renal categories was observed with increasing temperatures. This was most evident with AKI but was also observed with urolithiasis and UTIs, the latter especially with females. The results have implications for ongoing public health interventions aimed at preventing heat-related morbidity, by targeting individuals at greater risk of developing renal disease, including specific renal diseases, and improving community awareness of the risks with regards to heat and renal disease in Adelaide.

## Figures and Tables

**Fig. 6:**
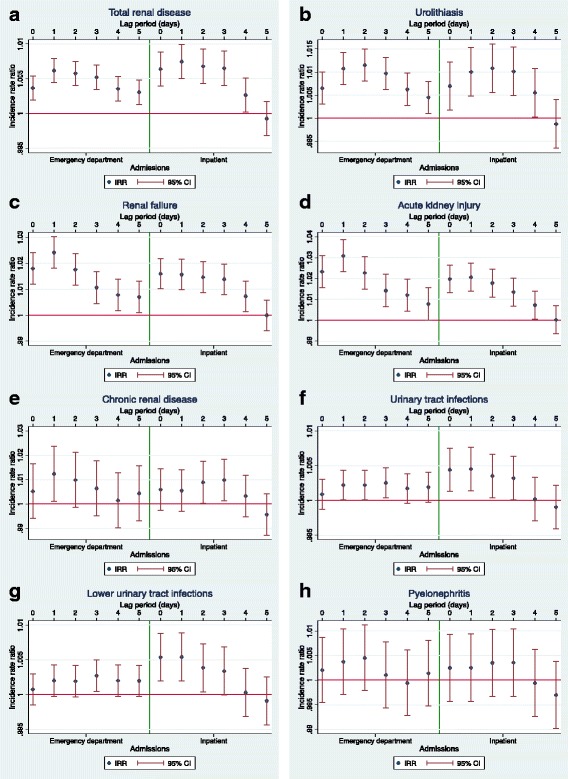
Incidence rate ratios (IRRs) for renal outcomes and daily maximum temperature for lag periods of 0–5 days. Graphs for incidence rate ratios (IRRs) for daily admissions for renal diseases and associated 95% confidence intervals (95% CI) in relation to daily maximum temperature per 1°C, during the warm season (October – March) in Adelaide from 1 July 2003 to 31 March 2014. Admissions are listed for each day over a lag period of 0 to 5 days. The left sides of the graphs represent emergency department admissions, and the right sides represent inpatient admissions. Graphs are in relation to (**a**); total renal disease (**b**); urolithiasis (**c**); renal failure (**d**); acute kidney injury (**e**); chronic kidney disease (**f**); urinary tract infections (**g**); lower urinary tract infections (**h**); pyelonephritis

**Fig. 7:**
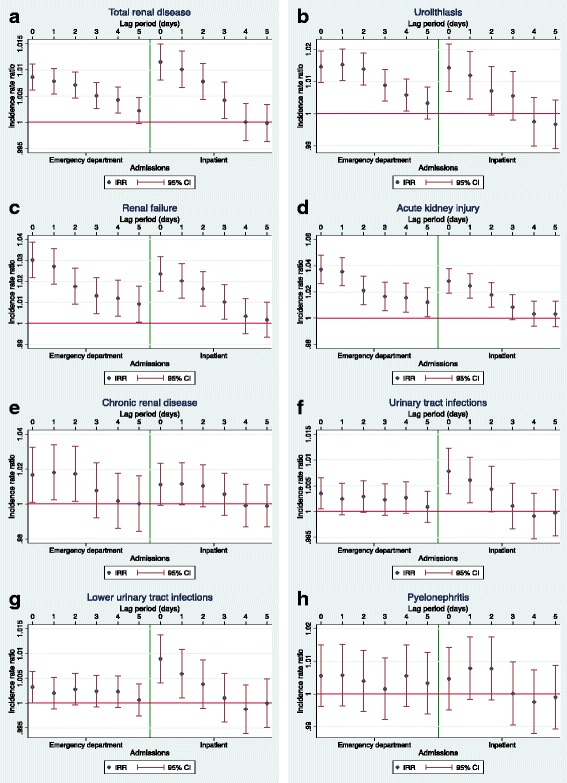
Incidence rate ratios (IRRs) for renal outcomes and daily minimum temperature for lag periods of 0–5 days. Graphs for incidence rate ratios (IRRs) for daily admissions for renal diseases and associated 95% confidence intervals (95% CI) in relation to daily minimum temperature per 1°C, during the warm season (October – March) in Adelaide from 1 July 2003 to 31 March 2014. Admissions are listed for each day over a lag period of 0 to 5 days. The left sides of the graphs represent emergency department admissions, and the right sides represent inpatient admissions. Graphs are in relation to (**a**); total renal disease (**b**); urolithiasis (**c**); renal failure (**d**); acute kidney injury (**e**); chronic kidney disease (**f**); urinary tract infections (**g**); lower urinary tract infections (**h**); pyelonephritis

**Fig. 8:**
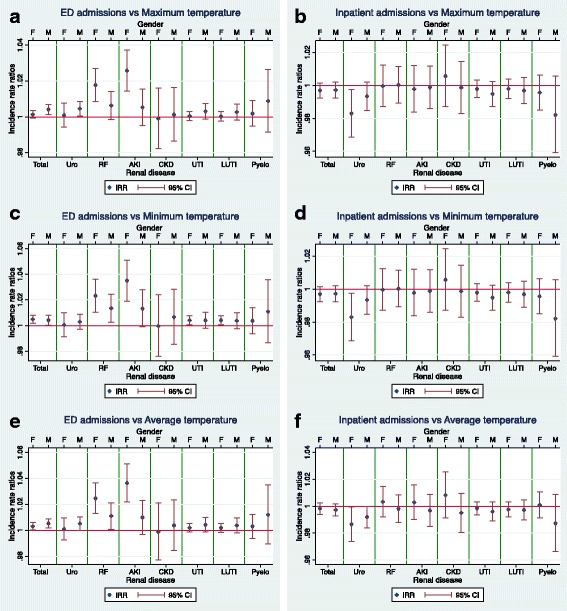
Incidence rate ratios (IRRs) for renal admissions and daily temperature stratified by gender. Graphs for incidence rate ratios (IRRs) for daily admissions for renal diseases and associated 95% confidence intervals (95% CI) in relation to daily temperature per 1°C, during the warm season (October – March) in Adelaide from 1 July 2003 to 31 March 2014. Results are shown for females (F) and males (M) separately. Graphs (**a**), (**c**) and (**e**) represent emergency department (ED) admissions, and graphs (**b**), (**d**) and (**f**) represent inpatient admissions. The temperature measures shown are maximum ((**a**) and (**b**)), minimum ((**c**) and (**d**)) and average ((**e**) and (**f**)). The renal categories are: total renal disease (Total); urolithiasis (Uro); renal failure (RF); acute kidney injury (AKI); chronic kidney disease (CKD); urinary tract infections (UTI); lower urinary tract infections (LUTI); pyelonephritis (Pyelo)

**Fig. 9:**
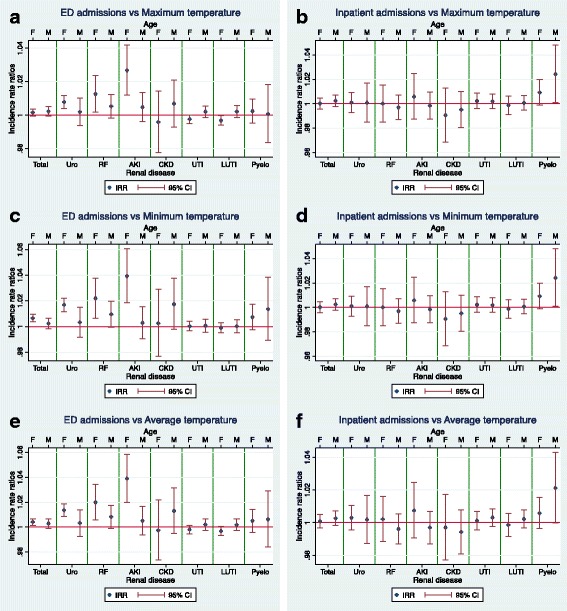
Incidence rate ratios (IRRs) for renal admissions and daily temperature stratified by age (<65 and 65+ years). Graphs for incidence rate ratios (IRRs) for daily admissions for renal diseases and associated 95% confidence intervals (95% CI) in relation to daily temperature, per 1°C, during the warm season (October – March) in Adelaide from 1 July 2003 to 31 March 2014. Results are shown for those <65 (<65) and ≥65 (65+) years. Graphs (**a**), (**c**) and (**e**) represent emergency department (ED) admissions, and graphs (**b**), (**d**) and (**f**) represent inpatient admissions. The temperature measures shown are maximum ((**a**) and (**b**)), minimum ((**c**) and (**d**)) and average ((**e**) and (**f**)). The renal categories are: total renal disease (Total); urolithiasis (Uro); renal failure (RF); acute kidney injury (AKI); chronic kidney disease (CKD); urinary tract infections (UTI); lower urinary tract infections (LUTI); pyelonephritis (Pyelo)

## Additional file

Additional file 1: Daily temperature data (maximum and minimum) from the Bureau of Meteorlogy from a monitoring station in Kent Town (station 023090), Adelaide. The data extends from July 2003 to March 2014. (XLS 556 kb)

## Appendix 1

**Table 4:** Poisson goodness-of-fit tests

Disease	Statistical test	Daily maximum temperature (1980 df)	
		ED	Inpatient
Total renal disease	Deviance goodness-of-fit statistic	2216.946	2271.521
	*P*-value	<0.001	<0.001
	Pearson goodness-of-fit statistic	2199.826	2244.133
	*P*-value	<0.001	<0.001
Urolithiasis	Deviance goodness-of-fit statistic	2290.703	2325.260
	*P*-value	<0.001	<0.001
	Pearson goodness-of-fit statistic	2150.742	2071.219
	*P*-value	0.004	0.075
Renal failure	Deviance goodness-of-fit statistic	2470.910	2387.224
	*P*-value	<0.001	<0.001
	Pearson goodness-of-fit statistic	2241.799	2133.986
	*P*-value	<0.001	0.008
AKI	Deviance goodness-of-fit statistic	2438.874	2407.545
	*P*-value	<0.001	<0.001
	Pearson goodness-of-fit statistic	2294.101	2171.447
	*P*-value	<0.001	0.002
CKD	Deviance goodness-of-fit statistic	1934.498	2279.640
	*P*-value	0.764	<0.001
	Pearson goodness-of-fit statistic	1906.882	2069.318
	*P*-value	0.878	0.079
UTI	Deviance goodness-of-fit statistic	2121.826	2131.721
	*P*-value	0.014	0.009
	Pearson goodness-of-fit statistic	2102.839	2079.242
	*P*-value	0.027	0.059
Lower UTI	Deviance goodness-of-fit statistic	2108.504	2168.087
	*P*-value	0.022	0.002
	Pearson goodness-of-fit statistic	2083.343	2103.063
	*P*-value	0.052	0.027
Pyelonephritis	Deviance goodness-of-fit statistic	2456.306	2257.211
	*P*-value	<0.001	<0.001
	Pearson goodness-of-fit statistic	2159.492	1949.885
	*P*-value	0.003	0.681

Poisson regression was used to estimate incidence rate ratios (IRRs) for daily emergency department (ED) and inpatient admissions for renal diseases in relation to an increase in daily maximum temperature per 1°C during the warm season (October – March) in Adelaide from 1 July 2003 to 31 March 2014. Deviance and Pearson goodness-of-fit tests were then applied to assess whether a Poisson model fits the data

The deviance and Pearson goodness-of-fit test show how well a Poisson model fits the data. Each test yields a test statistic, comparing the observed count to the expected count under a Poisson model. The test statistics and degrees of freedom (df) are used to generate P-values. A *P*-value of <0.05 indicates a poor fit of the Poisson model – most of the results indicated a poor fit

## Appendix 2

**Table 5:** Incidence rate ratios (IRRs) for renal outcomes and daily temperature for lag periods (0–5 days)

Temperature	Admission data	Disease	Lag period	IRR	95% CI			*P*-value
Maximum	ED	Total renal	0 days	1.004	1.002	–	1.005	<0.001
		disease	1 day	1.006	1.004	–	1.008	<0.001
			2 days	1.006	1.004	–	1.008	<0.001
			3 days	1.005	1.003	–	1.007	<0.001
			4 days	1.004	1.002	–	1.005	<0.001
			5 days	1.003	1.001	–	1.005	0.001
		Urolithiasis	0 days	1.006	1.003	–	1.010	<0.001
			1 day	1.011	1.007	–	1.014	<0.001
			2 days	1.011	1.008	–	1.015	<0.001
			3 days	1.010	1.006	–	1.013	<0.001
			4 days	1.006	1.003	–	1.010	<0.001
			5 days	1.004	1.001	–	1.008	0.012
		Renal failure	0 days	1.018	1.012	–	1.024	<0.001
			1 day	1.024	1.018	–	1.030	<0.001
			2 days	1.018	1.011	–	1.024	<0.001
			3 days	1.011	1.005	–	1.017	0.001
			4 days	1.008	1.002	–	1.014	0.012
			5 days	1.007	1.001	–	1.013	0.024
		AKI	0 days	1.023	1.016	–	1.031	<0.001
			1 day	1.031	1.023	–	1.039	<0.001
			2 days	1.023	1.015	–	1.030	<0.001
			3 days	1.014	1.006	–	1.022	<0.001
			4 days	1.012	1.004	–	1.020	0.002
			5 days	1.008	1.000	–	1.016	0.047
		CKD	0 days	1.005	0.994	–	1.016	0.365
			1 day	1.012	1.001	–	1.024	0.03
			2 days	1.010	0.999	–	1.021	0.085
			3 days	1.006	0.995	–	1.018	0.263
			4 days	1.001	0.990	–	1.013	0.802
			5 days	1.004	0.993	–	1.016	0.447
		UTI	0 days	1.001	0.999	–	1.003	0.411
			1 day	1.002	1.000	–	1.004	0.042
			2 days	1.002	1.000	–	1.004	0.043
			3 days	1.003	1.000	–	1.005	0.02
			4 days	1.002	1.000	–	1.004	0.114
			5 days	1.002	1.000	–	1.004	0.08
		Lower UTI	0 days	1.001	0.999	–	1.003	0.507
			1 day	1.002	1.000	–	1.004	0.078
			2 days	1.002	1.000	–	1.004	0.094
			3 days	1.003	1.000	–	1.005	0.018
			4 days	1.002	1.000	–	1.004	0.081
			5 days	1.002	1.000	–	1.004	0.086
		Pyelonephritis	0 days	1.002	0.995	–	1.009	0.547
			1 day	1.004	0.997	–	1.010	0.272
			2 days	1.005	0.998	–	1.011	0.184
			3 days	1.001	0.994	–	1.008	0.761
			4 days	0.999	0.993	–	1.006	0.868
			5 days	1.001	0.995	–	1.008	0.682
	Inpatient	Total renal	0 days	1.006	1.004	–	1.009	<0.001
		disease	1 day	1.007	1.005	–	1.010	<0.001
			2 days	1.007	1.004	–	1.009	<0.001
			3 days	1.006	1.004	–	1.009	<0.001
			4 days	1.003	1.000	–	1.005	0.035
			5 days	0.999	0.997	–	1.002	0.562
		Urolithiasis	0 days	1.007	1.002	–	1.012	0.009
			1 day	1.010	1.005	–	1.015	<0.001
			2 days	1.011	1.006	–	1.016	<0.001
			3 days	1.010	1.005	–	1.015	<0.001
			4 days	1.006	1.000	–	1.011	0.04
			5 days	0.999	0.993	–	1.004	0.631
		Renal failure	0 days	1.016	1.010	–	1.022	<0.001
			1 day	1.016	1.010	–	1.022	<0.001
			2 days	1.015	1.009	–	1.021	<0.001
			3 days	1.014	1.008	–	1.020	<0.001
			4 days	1.007	1.001	–	1.013	0.015
			5 days	1.000	0.994	–	1.006	0.993
		AKI	0 days	1.020	1.013	–	1.026	<0.001
			1 day	1.021	1.014	–	1.027	<0.001
			2 days	1.018	1.011	–	1.025	<0.001
			3 days	1.014	1.007	–	1.020	<0.001
			4 days	1.007	1.001	–	1.014	0.034
			5 days	1.000	0.994	–	1.007	0.955
		CKD	0 days	1.006	0.997	–	1.014	0.171
			1 day	1.005	0.997	–	1.014	0.208
			2 days	1.009	1.000	–	1.018	0.042
			3 days	1.010	1.001	–	1.018	0.023
			4 days	1.003	0.995	–	1.012	0.449
			5 days	0.996	0.987	–	1.004	0.317
		UTI	0 days	1.004	1.001	–	1.008	0.005
			1 day	1.005	1.001	–	1.008	0.004
			2 days	1.004	1.000	–	1.007	0.027
			3 days	1.003	1.000	–	1.006	0.044
			4 days	1.000	0.997	–	1.003	0.908
			5 days	0.999	0.996	–	1.002	0.548
		Lower UTI	0 days	1.005	1.002	–	1.009	0.002
			1 day	1.005	1.002	–	1.009	0.002
			2 days	1.004	1.000	–	1.007	0.028
			3 days	1.003	1.000	–	1.007	0.055
			4 days	1.000	0.997	–	1.004	0.866
			5 days	0.999	0.996	–	1.003	0.608
		Pyelonephritis	0 days	1.002	0.996	–	1.009	0.472
			1 day	1.003	0.996	–	1.009	0.47
			2 days	1.004	0.997	–	1.010	0.314
			3 days	1.004	0.997	–	1.010	0.307
			4 days	0.999	0.993	–	1.006	0.864
			5 days	0.997	0.990	–	1.004	0.386
Minimum	ED	Total renal	0 days	1.009	1.006	–	1.011	<0.001
		disease	1 day	1.008	1.005	–	1.010	<0.001
			2 days	1.007	1.005	–	1.010	<0.001
			3 days	1.005	1.003	–	1.008	<0.001
			4 days	1.004	1.002	–	1.007	0.001
			5 days	1.002	1.000	–	1.005	0.082
		Urolithiasis	0 days	1.015	1.010	–	1.020	<0.001
			1 day	1.015	1.010	–	1.020	<0.001
			2 days	1.014	1.009	–	1.019	<0.001
			3 days	1.009	1.004	–	1.014	<0.001
			4 days	1.006	1.001	–	1.011	0.022
			5 days	1.003	0.998	–	1.008	0.196
		Renal failure	0 days	1.030	1.022	–	1.039	<0.001
			1 day	1.027	1.019	–	1.036	<0.001
			2 days	1.018	1.009	–	1.026	<0.001
			3 days	1.013	1.005	–	1.022	0.002
			4 days	1.012	1.003	–	1.021	0.006
			5 days	1.009	1.001	–	1.018	0.036
		AKI	0 days	1.037	1.026	–	1.048	<0.001
			1 day	1.035	1.025	–	1.046	<0.001
			2 days	1.021	1.010	–	1.032	<0.001
			3 days	1.017	1.006	–	1.027	0.003
			4 days	1.016	1.005	–	1.027	0.005
			5 days	1.012	1.001	–	1.023	0.029
		CKD	0 days	1.017	1.001	–	1.033	0.036
			1 day	1.018	1.002	–	1.034	0.024
			2 days	1.017	1.002	–	1.033	0.031
			3 days	1.008	0.992	–	1.024	0.338
			4 days	1.002	0.986	–	1.018	0.83
			5 days	1.000	0.984	–	1.016	0.98
		UTI	0 days	1.004	1.000	–	1.007	0.023
			1 day	1.002	0.999	–	1.005	0.116
			2 days	1.003	1.000	–	1.006	0.06
			3 days	1.002	0.999	–	1.005	0.138
			4 days	1.003	1.000	–	1.006	0.082
			5 days	1.001	0.998	–	1.004	0.562
		Lower UTI	0 days	1.003	1.000	–	1.006	0.045
			1 day	1.002	0.999	–	1.005	0.216
			2 days	1.003	1.000	–	1.006	0.087
			3 days	1.002	0.999	–	1.006	0.142
			4 days	1.002	0.999	–	1.006	0.153
			5 days	1.001	0.997	–	1.004	0.706
		Pyelonephritis	0 days	1.006	0.996	–	1.015	0.245
			1 day	1.006	0.996	–	1.015	0.232
			2 days	1.004	0.995	–	1.013	0.41
			3 days	1.002	0.992	–	1.011	0.752
			4 days	1.006	0.996	–	1.015	0.244
			5 days	1.003	0.994	–	1.013	0.495
	Inpatient	Total renal	0 days	1.012	1.008	–	1.015	<0.001
		disease	1 day	1.010	1.007	–	1.014	<0.001
			2 days	1.008	1.004	–	1.011	<0.001
			3 days	1.004	1.001	–	1.008	0.018
			4 days	1.000	0.997	–	1.004	0.987
			5 days	1.000	0.996	–	1.003	0.942
		Urolithiasis	0 days	1.014	1.007	–	1.022	<0.001
			1 day	1.012	1.004	–	1.019	0.002
			2 days	1.007	1.000	–	1.015	0.062
			3 days	1.006	0.998	–	1.013	0.147
			4 days	0.997	0.990	–	1.005	0.514
			5 days	0.997	0.989	–	1.004	0.39
		Renal failure	0 days	1.024	1.015	–	1.032	<0.001
			1 day	1.020	1.012	–	1.029	<0.001
			2 days	1.016	1.008	–	1.025	<0.001
			3 days	1.010	1.002	–	1.019	0.016
			4 days	1.003	0.995	–	1.012	0.431
			5 days	1.002	0.993	–	1.010	0.695
		AKI	0 days	1.028	1.019	–	1.038	<0.001
			1 day	1.025	1.015	–	1.034	<0.001
			2 days	1.018	1.008	–	1.027	<0.001
			3 days	1.008	0.999	–	1.018	0.082
			4 days	1.003	0.994	–	1.013	0.513
			5 days	1.003	0.994	–	1.013	0.521
		CKD	0 days	1.011	0.999	–	1.023	0.068
			1 day	1.012	1.000	–	1.024	0.059
			2 days	1.010	0.998	–	1.022	0.089
			3 days	1.006	0.994	–	1.018	0.361
			4 days	0.999	0.987	–	1.011	0.884
			5 days	0.999	0.987	–	1.011	0.859
		UTI	0 days	1.008	1.003	–	1.012	<0.001
			1 day	1.006	1.002	–	1.011	0.007
			2 days	1.004	1.000	–	1.009	0.054
			3 days	1.001	0.997	–	1.006	0.633
			4 days	0.999	0.995	–	1.004	0.698
			5 days	1.000	0.995	–	1.004	0.903
		Lower UTI	0 days	1.009	1.004	–	1.014	<0.001
			1 day	1.006	1.001	–	1.011	0.017
			2 days	1.004	0.999	–	1.009	0.126
			3 days	1.001	0.996	–	1.006	0.677
			4 days	0.999	0.994	–	1.004	0.624
			5 days	1.000	0.995	–	1.005	0.986
		Pyelonephritis	0 days	1.005	0.995	–	1.014	0.346
			1 day	1.008	0.998	–	1.018	0.11
			2 days	1.008	0.998	–	1.017	0.114
			3 days	1.000	0.990	–	1.010	0.986
			4 days	0.998	0.988	–	1.007	0.62
			5 days	0.999	0.989	–	1.009	0.833
Average	ED	Total renal	0 days	1.007	1.004	–	1.009	<0.001
		disease	1 day	1.009	1.006	–	1.011	<0.001
			2 days	1.008	1.006	–	1.010	<0.001
			3 days	1.007	1.004	–	1.009	<0.001
			4 days	1.005	1.003	–	1.007	<0.001
			5 days	1.004	1.001	–	1.006	0.002
		Urolithiasis	0 days	1.012	1.007	–	1.016	<0.001
			1 day	1.015	1.011	–	1.020	<0.001
			2 days	1.016	1.011	–	1.020	<0.001
			3 days	1.012	1.007	–	1.016	<0.001
			4 days	1.008	1.003	–	1.012	0.001
			5 days	1.005	1.001	–	1.010	0.024
		Renal failure	0 days	1.028	1.020	–	1.036	<0.001
			1 day	1.032	1.024	–	1.040	<0.001
			2 days	1.022	1.014	–	1.030	<0.001
			3 days	1.014	1.007	–	1.022	<0.001
			4 days	1.012	1.004	–	1.020	0.004
			5 days	1.010	1.002	–	1.018	0.015
		AKI	0 days	1.035	1.025	–	1.045	<0.001
			1 day	1.041	1.031	–	1.051	<0.001
			2 days	1.028	1.018	–	1.038	<0.001
			3 days	1.019	1.009	–	1.029	<0.001
			4 days	1.017	1.007	–	1.027	0.001
			5 days	1.012	1.002	–	1.022	0.021
		CKD	0 days	1.011	0.997	–	1.026	0.125
			1 day	1.018	1.004	–	1.033	0.014
			2 days	1.016	1.001	–	1.030	0.034
			3 days	1.009	0.994	–	1.023	0.248
			4 days	1.002	0.987	–	1.017	0.784
			5 days	1.004	0.989	–	1.018	0.621
		UTI	0 days	1.002	0.999	–	1.005	0.121
			1 day	1.003	1.000	–	1.006	0.041
			2 days	1.003	1.000	–	1.006	0.03
			3 days	1.003	1.000	–	1.006	0.028
			4 days	1.003	1.000	–	1.005	0.063
			5 days	1.002	0.999	–	1.005	0.165
		Lower UTI	0 days	1.002	0.999	–	1.005	0.184
			1 day	1.003	1.000	–	1.006	0.085
			2 days	1.003	1.000	–	1.006	0.063
			3 days	1.003	1.000	–	1.006	0.026
			4 days	1.003	1.000	–	1.006	0.068
			5 days	1.002	0.999	–	1.005	0.203
		Pyelonephritis	0 days	1.004	0.995	–	1.013	0.364
			1 day	1.006	0.997	–	1.014	0.207
			2 days	1.005	0.997	–	1.014	0.215
			3 days	1.002	0.993	–	1.010	0.732
			4 days	1.002	0.993	–	1.011	0.67
			5 days	1.003	0.994	–	1.011	0.564
	Inpatient	Total renal	0 days	1.010	1.007	–	1.013	<0.001
		disease	1 day	1.011	1.007	–	1.014	<0.001
			2 days	1.009	1.006	–	1.012	<0.001
			3 days	1.007	1.004	–	1.010	<0.001
			4 days	1.002	0.999	–	1.005	0.171
			5 days	0.999	0.996	–	1.003	0.689
		Urolithiasis	0 days	1.012	1.005	–	1.019	0.001
			1 day	1.013	1.007	–	1.020	<0.001
			2 days	1.012	1.005	–	1.019	<0.001
			3 days	1.011	1.004	–	1.018	0.002
			4 days	1.004	0.997	–	1.011	0.297
			5 days	0.998	0.991	–	1.004	0.483
		Renal failure	0 days	1.023	1.016	–	1.031	<0.001
			1 day	1.022	1.014	–	1.029	<0.001
			2 days	1.019	1.012	–	1.027	<0.001
			3 days	1.016	1.008	–	1.024	<0.001
			4 days	1.008	1.000	–	1.015	0.053
			5 days	1.001	0.993	–	1.008	0.864
		AKI	0 days	1.029	1.020	–	1.037	<0.001
			1 day	1.028	1.019	–	1.036	<0.001
			2 days	1.022	1.014	–	1.031	<0.001
			3 days	1.015	1.006	–	1.024	0.001
			4 days	1.007	0.999	–	1.016	0.096
			5 days	1.001	0.993	–	1.010	0.751
		CKD	0 days	1.010	0.999	–	1.021	0.087
			1 day	1.010	0.998	–	1.021	0.092
			2 days	1.012	1.001	–	1.023	0.037
			3 days	1.011	1.000	–	1.022	0.058
			4 days	1.002	0.991	–	1.014	0.679
			5 days	0.996	0.985	–	1.007	0.466
		UTI	0 days	1.007	1.003	–	1.011	0.001
			1 day	1.006	1.002	–	1.010	0.002
			2 days	1.005	1.001	–	1.009	0.02
			3 days	1.003	0.999	–	1.007	0.129
			4 days	1.000	0.996	–	1.004	0.906
			5 days	0.999	0.995	–	1.003	0.663
		Lower UTI	0 days	1.008	1.004	–	1.013	<0.001
			1 day	1.007	1.003	–	1.012	0.002
			2 days	1.005	1.000	–	1.009	0.033
			3 days	1.003	0.999	–	1.008	0.154
			4 days	1.000	0.995	–	1.004	0.901
			5 days	0.999	0.995	–	1.004	0.735
		Pyelonephritis	0 days	1.004	0.995	–	1.013	0.378
			1 day	1.005	0.997	–	1.014	0.229
			2 days	1.006	0.997	–	1.015	0.168
			3 days	1.003	0.994	–	1.012	0.501
			4 days	0.998	0.990	–	1.007	0.724
			5 days	0.997	0.988	–	1.006	0.526

Estimated incidence rate ratios (IRRs) and associated 95% confidence intervals and *P*-values for daily admissions for renal diseases in relation to an increase in daily maximum temperature per 1°C over lag 0–5 days during the warm seasons (October – March) in Adelaide from 1 July 2003 to 31 March 2014. Both emergency department (ED) and inpatient admissions were included. The associated lag periods with the highest IRR value (rounded to 3 decimal figures) and are statistically significant are underlined

## Appendix 3

**Table 6:** Incidence rate ratios (IRRs) for renal outcomes and daily temperature stratified by gender

Temperature	Admission data	Disease	Gender	IRR	95% CI			*P*-value
Maximum	ED	Total renal disease	F	1.001	0.999	–	1.003	0.231
			M	1.004	1.001	–	1.007	0.003
		Urolithiasis	F	1.001	0.994	–	1.008	0.788
			M	1.005	1.001	–	1.009	0.026
		Renal failure	F	1.018	1.009	–	1.027	<0.001
			M	1.006	0.999	–	1.014	0.109
		AKI	F	1.026	1.014	–	1.037	<0.001
			M	1.005	0.995	–	1.015	0.305
		CKD	F	0.999	0.982	–	1.016	0.902
			M	1.001	0.986	–	1.016	0.873
		UTI	F	1.000	0.998	–	1.003	0.733
			M	1.003	0.999	–	1.007	0.169
		Lower UTI	F	1.000	0.998	–	1.003	0.863
			M	1.003	0.998	–	1.007	0.237
		Pyelonephritis	F	1.002	0.995	–	1.009	0.619
			M	1.009	0.992		1.026	0.319
Minimum	ED	Total renal disease	F	1.005	1.002	–	1.008	0.002
			M	1.004	1.000	–	1.008	0.036
		Urolithiasis	F	1.001	0.991	–	1.010	0.912
			M	1.003	0.997	–	1.009	0.327
		Renal failure	F	1.023	1.010	–	1.036	<0.001
			M	1.013	1.002	–	1.025	0.017
		AKI	F	1.035	1.019	–	1.051	<0.001
			M	1.013	0.999	–	1.028	0.069
		CKD	F	1.000	0.976	–	1.024	0.974
			M	1.007	0.985	–	1.028	0.542
		UTI	F	1.004	1.001	–	1.007	0.022
			M	1.004	0.998	–	1.010	0.199
		Lower UTI	F	1.004	1.000	–	1.008	0.029
			M	1.004	0.997	–	1.010	0.262
		Pyelonephritis	F	1.004	0.994		1.014	0.481
			M	1.011	0.987	–	1.036	0.383
Average	ED	Total renal disease	F	1.003	1.000	–	1.006	0.032
			M	1.005	1.002	–	1.009	0.004
		Urolithiasis	F	1.001	0.992	–	1.010	0.834
			M	1.005	1.000	–	1.010	0.057
		Renal failure	F	1.025	1.013	–	1.037	<0.001
			M	1.011	1.001	–	1.021	0.034
		AKI	F	1.036	1.022	–	1.051	<0.001
			M	1.010	0.997	–	1.023	0.138
		CKD	F	0.999	0.977	–	1.021	0.913
			M	1.004	0.984	–	1.023	0.708
		UTI	F	1.002	0.999	–	1.005	0.218
			M	1.004	0.999	–	1.010	0.143
		Lower UTI	F	1.002	0.998	–	1.005	0.282
			M	1.004	0.998	–	1.010	0.205
		Pyelonephritis	F	1.003	0.994	–	1.012	0.529
			M	1.012	0.990	–	1.035	0.297
Maximum	Inpatient	Total renal disease	F	1.000	0.996	–	1.003	0.778
			M	1.001	0.999	–	1.003	0.231
		Urolithiasis	F	0.992	0.983	–	1.002	0.139
			M	1.001	0.994	–	1.008	0.788
		Renal failure	F	1.004	0.995	–	1.013	0.347
			M	1.018	1.009	–	1.027	<0.001
		AKI	F	1.005	0.995	–	1.014	0.361
			M	1.026	1.014	–	1.037	<0.001
		CKD	F	1.007	0.994	–	1.020	0.289
			M	0.999	0.982	–	1.016	0.902
		UTI	F	0.999	0.996	–	1.003	0.710
			M	1.000	0.998	–	1.003	0.733
		Lower UTI	F	0.998	0.994	–	1.002	0.420
			M	1.000	0.998	–	1.003	0.863
		Pyelonephritis	F	1.003	0.996	–	1.011	0.400
			M	0.994	0.978	–	1.010	0.470
Minimum	Inpatient	Total renal disease	F	0.997	0.992	–	1.002	0.190
			M	0.997	0.992	–	1.002	0.256
		Urolithiasis	F	0.983	0.969	–	0.997	0.021
			M	0.993	0.985	–	1.002	0.135
		Renal failure	F	1.000	0.987	–	1.012	0.946
			M	1.000	0.989	–	1.012	0.958
		AKI	F	0.998	0.984	–	1.012	0.750
			M	0.999	0.986	–	1.012	0.852
		CKD	F	1.006	0.987	–	1.024	0.556
			M	0.999	0.983	–	1.015	0.860
		UTI	F	0.998	0.992	–	1.003	0.415
			M	0.995	0.987	–	1.002	0.183
		Lower UTI	F	0.998	0.992	–	1.004	0.494
			M	0.997	0.989	–	1.005	0.437
		Pyelonephritis	F	0.996	0.985	–	1.006	0.419
			M	0.982	0.959	–	1.006	0.133
Average	Inpatient	Total renal disease	F	0.998	0.994	–	1.003	0.429
			M	0.997	0.993	–	1.002	0.218
		Urolithiasis	F	0.987	0.974	–	1.000	0.045
			M	0.992	0.984	–	1.000	0.047
		Renal failure	F	1.003	0.992	–	1.015	0.567
			M	0.998	0.988	–	1.008	0.706
		AKI	F	1.003	0.990	–	1.016	0.658
			M	0.997	0.985	–	1.009	0.592
		CKD	F	1.008	0.991	–	1.025	0.341
			M	0.995	0.981	–	1.010	0.505
		UTI	F	0.998	0.994	–	1.003	0.533
			M	0.996	0.989	–	1.003	0.271
		Lower UTI	F	0.998	0.992	–	1.003	0.400
			M	0.997	0.990	–	1.005	0.458
		Pyelonephritis	F	1.001	0.991	–	1.011	0.855
			M	0.987	0.966	–	1.009	0.243

Estimated incidence rate ratios (IRRs) and associated 95% confidence intervals and *P*-values for daily admissions for renal diseases for males and females separately in relation to an increase in daily maximum temperature per 1°C during the warm seasons (October – March) in Adelaide from 1 July 2003 to 31 March 2014. Both emergency department (ED) and inpatient admissions were included

## Appendix 4

**Table 7:** Incidence rate ratios (IRRs) for renal outcomes and daily temperature stratified by age (<65 and 65+ years)

Temperature	Admission data	Disease	Age	IRR	95% CI			*P*-value
Maximum	ED	Total renal disease	<65	1.002	0.999	–	1.004	0.137
			65+	1.002	0.999	–	1.005	0.127
		Urolithiasis	<65	1.008	1.004	–	1.012	<0.001
			65+	1.002	0.994	–	1.010	0.650
		Renal failure	<65	1.013	1.002	–	1.024	0.023
			65+	1.005	0.998	–	1.012	0.143
		AKI	<65	1.027	1.012	–	1.042	0.000
			65+	1.005	0.996	–	1.014	0.287
		CKD	<65	0.996	0.978	–	1.015	0.660
			65+	1.007	0.993	–	1.021	0.336
		UTI	<65	0.998	0.995	–	1.000	0.067
			65+	1.002	0.999	–	1.006	0.245
		Lower UTI	<65	0.997	0.994	–	1.000	0.025
			65+	1.002	0.999	–	1.006	0.243
		Pyelonephritis	<65	1.002	0.995	–	1.010	0.510
			65+	1.001	0.984	–	1.018	0.937
Minimum	ED	Total renal disease	<65	1.007	1.004	–	1.010	<0.001
			65+	1.002	0.998	–	1.006	0.268
		Urolithiasis	<65	1.017	1.012	–	1.022	<0.001
			65+	1.003	0.992	–	1.015	0.581
		Renal failure	<65	1.022	1.007	–	1.038	0.005
			65+	1.010	0.999	–	1.020	0.064
		AKI	<65	1.039	1.019	–	1.061	<0.001
			65+	1.003	0.990	–	1.015	0.658
		CKD	<65	1.002	0.977	–	1.029	0.852
			65+	1.017	0.998	–	1.038	0.084
		UTI	<65	1.000	0.997	–	1.004	0.905
			65+	1.001	0.996	–	1.006	0.792
		Lower UTI	<65	0.999	0.995	–	1.003	0.629
			65+	1.000	0.995	–	1.005	0.968
		Pyelonephritis	<65	1.007	0.997	–	1.018	0.152
			65+	1.014	0.989	–	1.039	0.274
Average	ED	Total renal disease	<65	1.004	1.001	–	1.007	0.003
			65+	1.003	0.999	–	1.007	0.131
		Urolithiasis	<65	1.014	1.009	–	1.019	<0.001
			65+	1.003	0.993	–	1.014	0.555
		Renal failure	<65	1.020	1.006	–	1.034	0.006
			65+	1.008	0.999	–	1.018	0.076
		AKI	<65	1.039	1.020	–	1.059	<0.001
			65+	1.005	0.994	–	1.017	0.379
		CKD	<65	0.997	0.974	–	1.022	0.831
			65+	1.013	0.995	–	1.031	0.160
		UTI	<65	0.998	0.995	–	1.001	0.246
			65+	1.002	0.998	–	1.007	0.377
		Lower UTI	<65	0.997	0.993	–	1.000	0.089
			65+	1.002	0.997	–	1.007	0.431
		Pyelonephritis	<65	1.005	0.996	–	1.014	0.285
			65+	1.006	0.984	–	1.029	0.587
Maximum	Inpatient	Total renal disease	<65	1.001	0.998	–	1.004	0.587
			65+	1.002	0.999	–	1.005	0.229
		Urolithiasis	<65	1.003	0.997	–	1.009	0.334
			65+	1.002	0.991	–	1.013	0.754
		Renal failure	<65	1.002	0.992	–	1.013	0.657
			65+	0.997	0.990	–	1.004	0.388
		AKI	<65	1.006	0.993	–	1.019	0.389
			65+	0.997	0.990	–	1.005	0.479
		CKD	<65	1.001	0.986	–	1.017	0.881
			65+	0.996	0.986	–	1.006	0.419
		UTI	<65	1.000	0.996	–	1.005	0.887
			65+	1.003	0.999	–	1.007	0.185
		Lower UTI	<65	0.999	0.994	–	1.004	0.693
			65+	1.002	0.998	–	1.006	0.282
		Pyelonephritis	<65	1.002	0.995	–	1.010	0.540
			65+	1.013	0.997	–	1.030	0.115
Minimum	Inpatient	Total renal disease	<65	1.000	0.996	–	1.005	0.896
			65+	1.002	0.998	–	1.007	0.309
		Urolithiasis	<65	1.001	0.993	–	1.009	0.782
			65+	1.001	0.985	–	1.017	0.912
		Renal failure	<65	1.000	0.985	–	1.015	0.995
			65+	0.997	0.987	–	1.007	0.563
		AKI	<65	1.006	0.987	–	1.025	0.540
			65+	0.998	0.988	–	1.009	0.780
		CKD	<65	0.991	0.969	–	1.013	0.406
			65+	0.995	0.981	–	1.010	0.516
		UTI	<65	1.002	0.996	–	1.009	0.489
			65+	1.002	0.996	–	1.008	0.499
		Lower UTI	<65	0.999	0.991	–	1.006	0.749
			65+	1.001	0.995	–	1.007	0.812
		Pyelonephritis	<65	1.009	0.999	–	1.020	0.080
			65+	1.024	1.001	–	1.048	0.042
Average	Inpatient	Total renal disease	<65	1.001	0.997	–	1.005	0.667
			65+	1.003	0.998	–	1.007	0.222
		Urolithiasis	<65	1.003	0.996	–	1.010	0.439
			65+	1.002	0.987	–	1.017	0.805
		Renal failure	<65	1.002	0.988	–	1.016	0.762
			65+	0.996	0.987	–	1.005	0.404
		AKI	<65	1.007	0.991	–	1.025	0.390
			65+	0.997	0.987	–	1.007	0.554
		CKD	<65	0.997	0.977	–	1.017	0.772
			65+	0.994	0.981	–	1.008	0.399
		UTI	<65	1.001	0.995	–	1.007	0.675
			65+	1.003	0.998	–	1.008	0.249
		Lower UTI	<65	0.999	0.992	–	1.006	0.695
			65+	1.002	0.997	–	1.008	0.429
		Pyelonephritis	<65	1.006	0.996	–	1.015	0.231
			65+	1.021	1.000	–	1.043	0.051

Estimated incidence rate ratios (IRRs) and associated 95% confidence intervals and *P*-values for daily admissions for renal diseases in relation to an increase in daily maximum temperature per 1°C during the warm seasons (October – March) in Adelaide from 1 July 2003 to 31 March 2014. Patients are separated into two groups: one for patients aged <65 years old, another for patients aged ≥65 years old. Both emergency department (ED) and inpatient admissions were included

## Abbreviations

AKI: Acute kidney injury
CKD: Chronic kidney disease
ECF: Extracellular fluid
ED: Emergency department
ICD-10: International Classification of Diseases, 10th revision
IRR: Incidence rate ratio
LUTIs: Lower urinary tract infections
NB: Negative binomial
SA: South Australian
USA: United States of America
UTIs: Urinary tract infections
